# *Trichoderma*-Based Biostimulants Modulate Rhizosphere Microbial Populations and Improve N Uptake Efficiency, Yield, and Nutritional Quality of Leafy Vegetables

**DOI:** 10.3389/fpls.2018.00743

**Published:** 2018-06-05

**Authors:** Nunzio Fiorentino, Valeria Ventorino, Sheridan L. Woo, Olimpia Pepe, Armando De Rosa, Laura Gioia, Ida Romano, Nadia Lombardi, Mauro Napolitano, Giuseppe Colla, Youssef Rouphael

**Affiliations:** ^1^Department of Agricultural Sciences, University of Naples Federico II, Portici, Italy; ^2^CIRAM-Interdepartmental Center for Environmental Research, University of Naples Federico II, Naples, Italy; ^3^Task Force on Microbiome Studies, University of Naples Federico II, Naples, Italy; ^4^Department of Pharmacy, University of Naples Federico II, Naples, Italy; ^5^National Research Council, Institute for Sustainable Plant Protection, Portici, Italy; ^6^Department of Agricultural and Forestry Sciences, University of Tuscia, Viterbo, Italy

**Keywords:** *Eruca sativa* Mill., *Lactuca sativa* L., mineral composition, N uptake, *Trichoderma*, DGGE, microbial diversity

## Abstract

Microbial inoculants such as *Trichoderma*-based products are receiving great interest among researchers and agricultural producers for their potential to improve crop productivity, nutritional quality as well as resistance to plant pathogens/pests and numerous environmental stresses. Two greenhouse experiments were conducted to assess the effects of *Trichoderma*-based biostimulants under suboptimal, optimal and supraoptimal levels of nitrogen (N) fertilization in two leafy vegetables: Iceberg lettuce (*Lactuca sativa* L.) and rocket (*Eruca sativa* Mill.). The yield, nutritional characteristics, N uptake and mineral composition were analyzed for each vegetable crop after inoculation with *Trichoderma* strains *T. virens* (GV41) or *T. harzianum* (T22), and results were compared to non-inoculated plants. In addition, the effect of the *Trichoderma*-based biostimulants on microbes associated with the rhizosphere in terms of prokaryotic and eukaryotic composition and concentration using DGGE was also evaluated. *Trichoderma*-based biostimulants, in particular GV41, positively increased lettuce and rocket yield in the unfertilized plots. The highest marketable lettuce fresh yield was recorded with either of the biostimulant inoculations when plants were supplied with optimal levels of N. The inoculation of rocket with GV41, and to a lesser degree with T22, elicited an increase in total ascorbic acid under both optimal and high N conditions. *T. virens* GV41 increased N-use efficiency of lettuce, and favored the uptake of native N present in the soil of both lettuce and rocket. The positive effect of biostimulants on nutrient uptake and crop growth was species-dependent, being more marked with lettuce. The best biostimulation effects from the *Trichoderma* treatments were observed in both crops when grown under low N availability. The *Trichoderma* inoculation strongly influenced the composition of eukaryotic populations in the rhizosphere, in particularly exerting different effects with low N levels in comparison to the N fertilized plots. Overall, inoculations with *Trichoderma* may be considered as a viable strategy to manage the nutrient content of leafy horticulture crops cultivated in low fertility soils, and assist vegetable growers in reducing the use of synthetic fertilizers, developing sustainable management practices to optimize N use efficiency.

## Introduction

Italy is the European leader for leafy vegetable production with about 15,000 ha and 150 kilo-tons *per annum* in protected greenhouses^1^ that are mainly produced in Campania region (southern Italy) and in northern regions of Lombardia and Veneto. Among the leafy vegetables destined as fresh cut produce, lettuce (*Lactuca sativa* L.) and rocket (*Eruca sativa* Mill.) have been gaining prominence in the national and international vegetable markets (Colonna et al., 2016).

Leafy vegetable crop production relies heavily on nitrate (NO_3_^-^) availability which constitutes the most important source of nitrogen (N) (Colla et al., 2010, 2011). Roughly, half of the N fertilizer applied is utilized by the vegetable crops, however, the remainder is lost by leaching to the soil, which can contribute to surface and groundwater contamination (Tilman et al., 2002). The search for strategies to improve agricultural practices, aimed at increasing uptake efficiency without affecting crop productivity, represents a strong stimulus for researchers, extension specialists as well as vegetable growers (Colla et al., 2018). Over the past three decades many efforts have been employed to enhance crop nitrogen use efficiency (NUE), through plant breeding programs and biotechnological approaches, however, with limited success and economic benefits due to the complexity of the genetic traits involved (Xu et al., 2012).

The use of plant biostimulants, which include organic and inorganic natural substances (i.e., humic acids, protein hydrolysates, seaweed extracts, and silicon) as well as beneficial microorganisms (i.e., mycorrhizal fungi, *Trichoderma* spp. and plant growth promoting rhizobacteria) to enhance nutrient uptake and crop production could be considered as a sustainable and environmentally friendly approach to secure yield stability under low-input conditions (Ventorino et al., 2014; Colla et al., 2015a, 2017; du Jardin, 2015; Lorito and Woo, 2015; Rouphael et al., 2015; Viscardi et al., 2016). According to López-Bucio et al. (2015) *Trichoderma* have gained importance as microbial plant biostimulants in horticulture. In the past, *Trichoderma-*based products were particularly noted as successful biological control agents for contrasting plant pathogens, mainly phytopathogenic fungi, as well as inducing resistance to biotic stresses (Harman, 2000; Harman et al., 2004; Hermosa et al., 2012; Woo et al., 2014; Lorito and Woo, 2015). However, in addition to their biopesticide activity, some *Trichoderma* strains have been proven to have a biostimulant activity, plant growth promotion, improved yield and nutritional quality, as well as mitigating the detrimental effect of abiotic stresses (Lorito et al., 2010; Hermosa et al., 2012; López-Bucio et al., 2015). Therefore, it is not surprising that *Trichoderma* are found as successful beneficial microbial biological agents, also present as active ingredients in over 200 agricultural products such as biopesticides, biofertilizers, bio-growth enhancers and biostimulants marketed worldwide for conventional and organic agricultural production (Woo et al., 2014).

In a recent review, López-Bucio et al. (2015) reported that the mechanism of stimulation by *Trichoderma* involves a multilevel root–shoot communication. The phytostimulation effect of *Trichoderma* applications has been attributed to several direct and indirect effects on plants, including the release of substances with auxin activity (i.e., indole-3-acetaldehyde, indole-3-carboxaldehyde, and indole-3-ethanol), small peptides as well as volatile organic compounds, which improve root system architecture (total root length, density, and branching) and assimilation/solubilization of macronutrients (P) and micronutrients (Fe, Mn, and Zn), thus boosting plant growth and crop productivity (Harman, 2000; Howell, 2003; Harman et al., 2004; Contreras-Cornejo et al., 2009, 2011; Lorito et al., 2010; Hermosa et al., 2012; Lorito and Woo, 2015; Colla et al., 2015b,c; Rouphael et al., 2017a). Furthermore, endophytic fungi including *Trichoderma* spp. interact with other members of the microbial community in the plant rhizosphere and therefore it is important to assess the ecological impact of different soil managements and microbial-based biostimulant on soil ecosystem and in particular on quantitative and qualitative microbial populations (Mar Vázquez et al., 2000; Lace et al., 2015).

Although studies on the biostimulant role of *Trichoderma* on eliciting plant growth and yield as well as on enhancing tolerance to environmental stresses such as salinity and drought (López-Bucio et al., 2015) have been found, nothing is known about the beneficial responses on *Trichoderma* application on leafy vegetables grown under different N inputs. Moreover, the application of inoculants, chemical fertilizers or a combination of organic and chemical strategies, could modulate the microbial communities in the plant rhizosphere (Mar Vázquez et al., 2000), with an ecological impact on agro-ecosystem that can be investigated by culture-independent molecular techniques (Belyaeva et al., 2012; Ventorino et al., 2013, 2016; Gupta et al., 2014, 2016).

The aim of the current study was to determine the effect of different *Trichoderma*-based biological treatments (*T. harzianum* strain T22 and *T. virens* strain GV41) on the quantitative and qualitative characteristics, including the chemical composition of two important leafy vegetables, lettuce and rocket, cultivated in different fertilization conditions corresponding to suboptimal, optimal, and supraoptimal N levels for each crop. Furthermore, analysis was conducted to evaluate the effect of the N fertilization and microbial-biostimulant applications on the microbes associated to the rhizosphere of the two horticultural crops in terms of prokaryotic and eukaryotic composition and concentration. The knowledge gained from this research will shed light on the mode of action of *Trichoderma* strains under different N availability and will permit the development of sustainable farming strategies to cultivate highly productive leafy vegetables using reduced amounts of synthetic N fertilizer.

## Materials and Methods

### Plant Material, Experimental Conditions, and Design

Two consecutive experiments were conducted in the 2016 winter-summer growing season, the first on lettuce (*Lactuca sativa* L. var. Iceberg cv. ‘Silvinas’; Rijk Zwaan, Bologna, Italy) from February 2nd to March 31st (Experiment 1), and the second on rocket (*Eruca sativa* Mill.) from June 13th to July 11th (Experiment 2). Experiments were conducted in a unheated polyethylene greenhouse located at the University of Naples Federico II, Portici (NA), south Italy (40° 49’N, 14° 15’E; 72 m a.s.l.). The soil was a sandy loam (76% sand, 17% silt, 7% clay), with a pH of 6.9, electrical conductivity of 0.6 mS cm^-1^, organic matter of 1.25% (w/w), C:N of 10.8, total N at 0.11%, carbonates at 0.3%, NO_3_-N and NH_4_-N at 108 and 13 mg kg^-1^, respectively, P at 38 mg kg^-1^, and exchangeable K at 980 mg kg^-1^.

The test conditions for each horticultural crop consisted of three fertilization regimes and three microbial inoculations. Suboptimal (Low N), optimal (Opt N) and supraoptimal (High N), fertilization levels were applied to the two crops corresponding to 0, 90, and 180 kg N ha^-1^for lettuce, and 0, 60, and 120 kg N ha^-1^for rocket. Microbial-based biostimulant treatments included *Trichoderma harzianum* strain T22 (T22) and *Trichoderma virens* strain GV41 (GV41), as well as a control (NoT).

In both experiments, nine treatments from the factorial combination of the above-described fertilization rates and microbial applications were arranged in randomized complete block split-plot design (N fertilization as the main factor and *Trichoderma* inoculation as sub-factor) with three replicates for a total of 27 experimental plots. The area of each experimental plot measured 3.5 m^2^, containing an expected plant density of 11 plants m^-2^ for lettuce and 3,000 seeds m^-2^ for rocket.

No P and K fertilization was performed due to the high content of these macronutrients found in the soil samples examined. The N was applied as ammonium nitrate (34%) by fertigation, in a drip irrigation system with in-line emitters at 35 cm distances, and an emitter flow rate of 3.3 L h^-1^ was adopted for lettuce, whereby the total N amount was allocated in 3 weekly applications starting 10 days after transplanting (DAT). Instead for rocket, N fertigation was performed using a sprinkler irrigation system, with applications at 3 and 8 days after seeding (DAS), thus distributing the total N amount into two equal doses. In both experiments, the NO_3_^-^ concentration in the water source used for the irrigation was lower than 50 mg L^-1^; therefore the N concentration coming from irrigation water was considered negligible. Throughout the growing cycle, pathogens and pests were controlled according to standard crop protection practices adopted by commercial leafy vegetable growers in Italy.

### Preparation of Fungal Biostimulant Inoculum

*Trichoderma harzianum* strain T22 and *T. virens* strain GV41, were obtained from the microbe collection of the Department of Agricultural Sciences, University of Naples Federico II, Portici. Fungi were grown on Potato Dextrose Agar (PDA, HiMedia Mumbai, India), at room temperature (25°C), with light (16 h day/8 h night), until sporulation. Conidial spores were collected in sterile water, then a 50 ml spore suspension (concentration of 1 × 10^6^ spores ml^-1^) was used as a starter inoculum for solid-state fermentation on sterile rice (500 g), in breathable bags (Microsac, Nevele, Belgium), then incubated at 25°C with light (16 h day/8 h night) in a growth chamber. After 7 days the spores were collected by washing the colonized rice with sterile water. The final concentration of the spore suspension was adjusted to 1 × 10^7^ spores ml^-1^, then used for the microbial-biostimulant applications in both greenhouse trials.

Lettuce seedlings were treated with the *Trichoderma* inoculum by using a root dip method. At the time of transplant, the styrofoam trays containing the seedlings were submerged in the liquid spore suspension for 10 min, in order to completely wet the roots. Plant trays were drained of excess liquid, the plant-plug was removed from the tray, then transplanted to pre-bored holes in the soil. Each plant was watered at the base with 25 ml of the spore suspension. The microbial-biostimulant treatment was repeated at 24 DAT, by watering 50 ml plant^-1^ of the spore suspension.

For the rocket experiment, the *Trichoderma* inoculations were applied as a seed-coating treatment, using a 1 × 10^8^ spores ml^-1^ spore suspension to uniformly cover the seed surface, then left to air-dry, and hand-seeded to the prepared soil at approximate concentration of 3,000 seeds m^-2^.

### Evaluation of Lettuce and Rocket Productivity

Lettuce plants were harvested 60 DAT in order to evaluate the total and marketable yields, sampling in one square meter quadrats from the center of each experimental plot. Total and marketable yield of rocket was determined at 30 DAS following the same procedure as for lettuce. Vegetative material of both lettuce and rocket was weighed at harvest to obtain the fresh weight, then dried at 80°C for about 72 h until achieving constant weight, and weighed again to determine the dry matter content of the plant biomass. A sub-sample of the dried leaf tissues was collected for the ion analyses. To determine the amount of ascorbic acid (AsA) in lettuce and rocket, fresh leaves were frozen in liquid nitrogen and stored at -80°C until used.

### Analysis of Total Ascorbic Acid Content

The total ascorbic acid in lettuce and rocket leaf extracts, defined as ascorbic acid and dehydroascorbate acid, was determined by an assay based on the reduction of Fe^3+^ to Fe^2+^ by AsA and the spectrophotometric detection of Fe^2+^ complexes with 2,2-dipyridyl (Kampfenkel et al., 1995). Dehydroascorbate is reduced to ascorbic acid by pre-incubation of the sample with dithiothreitol. The absorbance of the solution was measured at 525 nm in a spectrophotometer Hach DR 2000 (Hach Co., Loveland, CO, United States), and data were expressed as mg ascorbic acid on 100 g fresh weight.

### Leaf Ion Analyses

Dried lettuce or rocket leaf tissues were ground separately in a Wiley mill (IKA, MF10.1, Staufen, Germany) and passed through 0.5 mm sieve, then the homogenized plant tissues were used for ion analyses.

For the cations (K^+^ and Ca^2+^) and anions (NO_3_^-^ and PO_4_^3-^) analysis, 250 mg of dried material was extracted in 50 ml of ultrapure water using a shaking water bath (ShakeTemp SW22, Julabo, Seelbach, Germany) at 80°C for 10 min, as described by Rouphael et al. (2017b,c). Briefly, the mixture was centrifuged at 6,000 rpm for 10 min (R-10 M, Remi Elektrotechnik Limited, India), then filtered through a 0.20 μm filter paper (Whatman International Ltd., Maidstone, United Kingdom). The monovalent and bivalent cations were separated by ion chromatography (ICS-3000, Dionex, Sunnyvale, CA, United States) and quantified with an electrical conductivity detector. A conductivity detector with IonPac CG12A (4 × 250 mm, Dionex, Corporation) guard column and IonPac CS12A (4 × 250 mm, Dionex, Corporation) analytical column were used for the analysis of the monovalent and bivalent cations, whereas for nitrate and phosphate an IonPac AG11-HC guard (4 × 50 mm) column and IonPac AS11-HC analytical column (4 × 250 mm) were used.

### Analysis of Soil Minerals

Soil nitrogen (total N) concentration was assessed after mineralization with sulfuric acid (96%, Carlo Erba Reagents, Milan, Italy) in the presence of potassium sulfate and a low concentration of copper by the Kjeldahl method (Bremner, 1965). Mineral N was determined spectrophotometrically (FIAstar 5000 Analyzer, FOSS analytical Denmark) on soil extracts. Gas semi-permeable membrane method (ISO11732 procedure^2^) was carried out for NH_4_-N, while the sulfanilamide-naphtylethylendiamine dihydrochloride method was carried out to analyze NO_3_-N after nitrate to nitrite reduction with a copper–cadmium column (ISO 13395 procedure^3^).

### Determination of Fungal Concentration in the Rhizo-Soil

At the time of harvest for experiments 1 and 2, soil samples were collected from the plant rhizosphere and the surrounding soil, from each treatment plot. Detection and quantification of fungal colony forming units (CFU) was conducted using standard soil microbial plating techniques by serial dilutions. The total number of fungal CFU were estimated on Rose Bengal-Chloramphenicol agar (HiMedia Pvt. Ltd., Mumbai, India) supplemented with 0.1% (v/v) Igepal (Sigma-Aldrich, Milan, Italy). One gram of soil was added to 100 ml of water and left in vigorous orbital agitation for 10 min; then 10-fold serial dilutions of the soil suspension were performed. A 100 μl aliquot of each soil dilution was spread on the surface of the solid media and plated in three replicates. The CFU of fungi were counted after incubation for 3–7 days at 25°C.

### Molecular Characterization of Soil Microbes

Total DNA was extracted from soil samples using a Fast DNA SPIN Kit for Soil (MP Biomedicals, Illkirch, France) according to the manufacturer’s instructions.

The primers V3f (5′-CCTACGGGAGGCAGCAG-3′) and V3r (5′-ATTACCGCGGCTGCTGG-3′) (Muyzer et al., 1993) were employed for prokaryotic DGGE analysis. The primers NL1 (5′-GCATATCAATAAGCGGAGGAAAAG-3′) (Kurtzman and Robnett, 1998) and LS2 (5′-ATTCCCAAACAACTCGACTC-3′) (Cocolin et al., 2000) of the 26S rRNA gene were used to analyze the eukaryotic population. As described by Muyzer et al. (1993), a GC clamp was added to forward primers. The PCR mixture and conditions for both amplifications were performed according to Ventorino et al. (2016). DGGE analyses were performed in a polyacrylamide gel [8% (wt/vol) acrylamide-bisacrylamide (37:5:1)] with a denaturing gradient of 30–60% using a Bio-Rad DCode Universal Mutation System (Bio-Rad Laboratories, Milan, Italy) as previously described (Ventorino et al., 2018).

### Statistical Analysis

Experimental data were statistically analyzed by two-way analysis of variance using the SPSS 21 software package. To separate treatment means within each measured parameter, Least Significant Difference (LSD) test was performed at a significance level of *p* ≤ 0.05. Phoretix 1 advanced version 3.01 software (Phoretix International Limited, Newcastle upon Tyne, England) was used to automatically detect the DGGE bands, matching bands were determined, then a cluster analysis was performed as previously indicated by Ventorino et al. (2013). The correlation matrix of the band patterns was performed by using the method described by Saitou and Nei (1987). Finally, the percentage of similarity (S) of the microbial community was estimated by analyzing the resulting matrix using the average linkage method in the cluster procedure of Systat 5.2.1.

## Results

### Fungal Concentration in the Soil

In general, the total fungal concentration (CFU) in rhizo-soils (including also the applied *Trichoderma* inoculum) of the two leafy vegetables was species-dependent, and also influenced by the nitrogen fertilizer applications as well as by the strains of *Trichoderma* used. In the absence of *Trichoderma* inoculation, the different N fertilizations did not modify the fungal CFU in lettuce rhizo-soil, whereas a significant reduction (by 46%) was observed only with the high N fertilization in rocket rhizo-soil. The overall CFU of fungi isolated from the lettuce soils ranged from 1.5 × 10^6^ to 1.7 × 10^7^ CFU g^-1^ of soil. Interestingly, in the unfertilized conditions, the highest number of fungal colonies in lettuce was observed in the treatments with GV41 (1.7 × 10^7^ CFU g^-1^ of soil), followed by NoT (1.0 × 10^7^ CFU g^-1^ of soil) and finally with plants inoculated with T22 (3.6 × 10^6^ CFU g^-1^ of soil). Moreover, under optimal N conditions, the fungal concentration did not differ between NoT and T22 treatments (1.3 × 10^7^ CFU g^-1^ of soil), which were significantly higher than that of GV41 (1.8 × 10^6^ CFU g^-1^ of soil). Under High N levels, the overall fungal presence was relatively high (1.5 × 10^7^ CFU g^-1^ of soil in NoT), and the greatest concentration among the inoculated plots was observed with GV41 (9.2 × 10^6^ CFU g^-1^ of soil) compared to the T22 (1.5 × 10^6^ CFU g^-1^ of soil) treatment.

The overall fungal concentration in rocket rhizo-soils, for all N fertilizer and biostimulant treatments was 10-fold less than those observed in the experiment conducted on the lettuce, with counts ranging from 1.6 × 10^5^ to 6.5 × 10^5^ CFU g^-1^ of soil. The fungal CFUs in both inoculated treatments were not different from the untreated control (NoT), at both low N (3.9 × 10^5^ CFU g^-1^ of soil) and optimal N (3.9 × 10^5^ CFU g^-1^ of soil) levels of fertilization. Nevertheless, there was an increased concentration of fungi found with treatments of T22 in Opt N (5.3 × 10^5^ CFU g^-1^ of soil), and with both biostimulants under the High N (average value of 5.9 × 10^5^ CFU g^-1^ of soil) fertilization regime. Finally, the response of the fungal populations to inoculations with either *Trichoderma*, in the High N fertilization regime, were always greater than the CFU found in the uninoculated control treatment (1.6 × 10^5^ CFU g^-1^ of soil), with GV41 exhibiting higher fungal concentrations (6.5 × 10^5^ CFU g^-1^ of soil) than T22 (5.3 × 10^5^ CFU g^-1^ of soil).

### Crop Yield and Nutritional Quality

**FIGURE 1:**
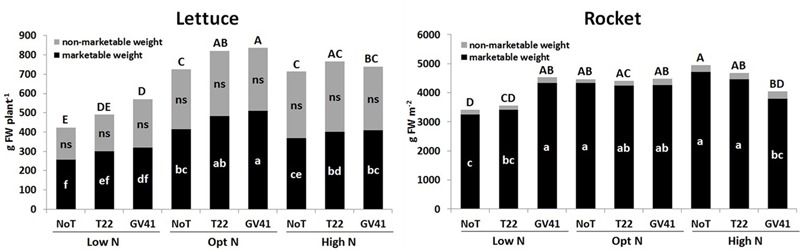
Total, marketable and non-marketable yield of lettuce and rocket as affected by N fertilization doses and *Trichoderma* inoculation. Low N, non-fertilized conditions (low N availability) for both crops; Opt N, optimal N fertilization, amounting to 90 kg N ha^-1^ and 60 kg N ha^-1^ for lettuce and rocket, respectively; High N, supraoptimal N fertilization, amounting to 180 and 120 kg N ha^-1^ for lettuce and rocket, respectively; NoT, non-inoculated control; T22, *T. harzianum* strain T22; GV41, *T. virens* strain GV41. Different letters indicate different means according to LSD test (*P* < 0.05). Lower cases are referred to the marketable weight, while upper cases are referred to the total weight production.

No visible chlorosis and/or necrosis symptoms were observed in both leafy vegetables a consequence of *Trichoderma* inoculation with T22 and GV41 (**Supplementary Figure S1**). *Trichoderma-*based biostimulants positively affected both lettuce and rocket yield in the unfertilized plots (Low N), but the effect of the fungal inocula on the production of the leafy vegetable crops was variable when N fertilizer was applied (**Figure 1**). The highest marketable lettuce fresh yield was recorded with both biostimulant inoculations when plants were supplied with the optimal N dose (90 kg N ha^-1^), but no significant differences were noted between the two *Trichoderma* strains (496 g FW plant^-1^, on the average). Lettuce grown in absence of N fertilization demonstrated a significant yield increase when inoculated with GV41 (by 34% and 24% for total and marketable weight, respectively), and a moderate increase with T22 (by 16% and 17% for total and marketable weight, respectively).

No microbial-biostimulant effect was recorded for rocket under optimal N supply (60 kg N ha^-1^), with an average marketable yield of 4,275 g m^-2^, while the GV41 treatment increased total yield of rocket by 33% in comparison to the non-inoculated (NoT) control in non-fertilized plots (**Figure 1**).

Crop productivity for both leafy vegetables under the excess N fertilization regime (180 kg N ha^-1^ and 120 kg ha^-1^ for lettuce and rocket, respectively) was similar to that observed under the optimal N treatments. Interestingly, in the High N condition, a reduction in lettuce yield was noted with the GV41 inoculation (by 11% and 19% for total and marketable yields, respectively) in comparison to Opt N. Similarly, with rocket grown under High N conditions, the GV41 biostimulant also significantly reduced yield in comparison to NoT and to T22 treatments.

**FIGURE 2:**
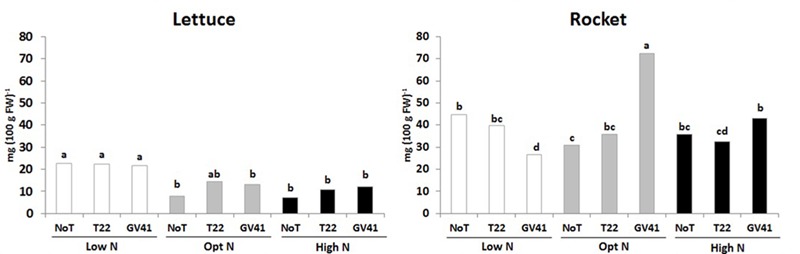
Ascorbic acid content in aboveground lettuce and rocket tissues as affected by N fertilization doses and *Trichoderma* inoculation. Low N, non-fertilized conditions (low N availability) for both crops; Opt N, optimal N fertilization, amounting to 90 kg N ha^-1^ and 60 kg N ha^-1^ for lettuce and rocket, respectively; High N, supraoptimal N fertilization, amounting to 180 and 120 kg N ha^-1^ for lettuce and rocket, respectively; NoT, non-inoculated control; T22, *T. harzianum* strain T22; GV41, *T. virens* strain GV41. Different letters indicate different means according to LSD test (*P* < 0.05).

In both leafy vegetables the total ascorbic acid content (including ascorbic and dehydroascorbic acid), which constitute and important component of functional quality of horticultural crops (Kyriacou and Rouphael, 2018), was significantly influenced by the *Trichoderma*-based biostimulants, N availability rate and their interaction (**Figure 2**). For instance, the total ascorbic acid content in lettuce was relatively low, ranging from 7.3 to 22.7 mg 100 g^-1^ FW, whereas the total ascorbic acid content in rocket was much greater, ranging from 26.4 to 72.7 mg 100 g^-1^ FW (**Figure 2**). In the lettuce experiment, the highest values of total ascorbic acid was recorded in unfertilized treatment irrespective of *Trichoderma* inoculation. In the case of rocket, the highest total ascorbic acid content was recorded in plants inoculated with GV41 at the optimal N level (**Figure 2**). Our results also demonstrated that inoculation with GV41 under both N fertilization treatments incurred a significant increase in total ascorbic acid compared with T22 and NoT plants, whereas an opposite trend was observed in unfertilized treatment (**Figure 2**).

### Nitrate Content and Total N Uptake

Neither N fertilization rate nor microbial-based biostimulant had a significant effect on nitrate content in either lettuce (average 1,614.2 mg kg^-1^ fw) or rocket (average 3,251.2 mg kg^-1^ fw). The nitrate content found in both leafy vegetables was below the maximum limit established by the European Commission regulations N° 1881/2006 and 1258/2011 for these two horticultural commodities: 2,000 mg kg^-1^ fw for lettuce and 6,000–7,000 mg kg^-1^ fw for rocket.

**FIGURE 3:**
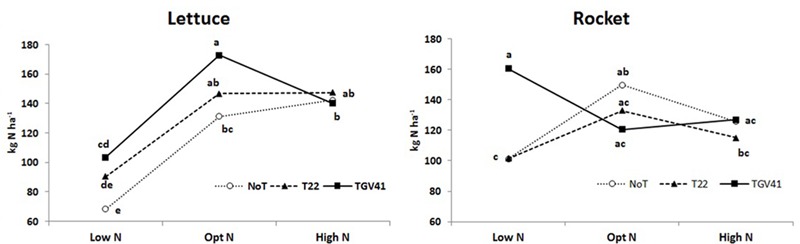
Total N uptake for lettuce and rocket as affected by different N doses and *Trichoderma* inoculation. Low N, non-fertilized conditions (low N availability) for both crops; Opt N, optimal N fertilization, amounting to 90 kg N ha^-1^ and 60 kg N ha^-1^ for lettuce and rocket, respectively; High N, supraoptimal N fertilization, amounting to 180 and 120 kg N ha^-1^ for lettuce and rocket, respectively; NoT, non-inoculated control; T22, *T. harzianum* strain T22; GV41, *T. virens* strain GV41. Different letters indicate different means according to LSD test (*P* < 0.05).

N-uptake in plants from unfertilized treatments was significantly greater in rocket (101 kg N ha^-1^) than in lettuce (68 kg N ha^-1^), and the microbial-based biostimulant effect under different N fertilization rates exhibited different responses in the two tested leafy vegetables. In absence of external N inputs, inoculation with GV41 permitted a higher uptake of native mineral N (by 51% and 59% in lettuce and rocket, respectively), whereas the inoculation with T22 treatment increased N uptake (by 33%) only in lettuce (**Figure 3**). Furthermore, inoculation with GV41 and T22 strains under optimal N conditions incurred a significant increase in lettuce N uptake by 32% and 12%, respectively, whereas the influence of *Trichoderma* inoculation in rocket was minimal or not significant (**Figure 3**). Finally, under High N fertilization the effect of microbial-based biostimulants on N uptake of lettuce and rocket was strongly limited (**Figure 3**). Soil mineral N content (in the 0–20 cm top layer) measured at lettuce harvest was not significantly affected by either biostimulant or fertilizer applications. Nevertheless, a tendency to higher soil mineral N content in the fertilized treatments (24 and 25 ppm for Opt N and High N, respectively) was recorded in comparison to the unfertilized control (20 ppm on the average). No differences were detected between Low N and fertilized treatments for rocket, with an average soil mineral N content of 12 ppm in the soil.

### Ion Content in Leaves

The K^+^ and Ca^2+^ and in particular, the PO_4_^3-^ concentrations in lettuce were highly influenced by N fertilization, *Trichoderma*-based biostimulant and the interaction (**Table 1**). The highest phosphate and calcium concentrations were recorded on lettuce plants inoculated with GV41 under Opt N (**Table 1**). Independent to N fertilization level, the greatest mineral accumulation (phosphate, potassium, and calcium) was observed in leaves of lettuce plants inoculated with GV41, then T22 (for PO_4_^3-^). Furthermore, in lettuce, the concentration of phosphate and potassium significantly increased in correspondence to an increase in the fertilization dose (from 0 to 180 kg N ha^-1^), with no significant differences noted between the Opt N and High N doses.

**Table 1:** Analysis of variance and mean comparisons for mineral concentration of lettuce and rocket leaves grown with different N fertilization dose-sand inoculated with *Trichoderma*-based biostimulants.

Treatments	Lettuce			Rocket		
	PO_4_^3-^	K^+^	Ca^2+^	PO_4_^3-^	K^+^	Ca^2+^
	g kg^-1^ (d.w.)			g kg^-1^ (d.w.)		
NoT	2.9 b	67.6 b	8.3 b	11.0	60.1	24.9 b
T22	4.1 a	75.5 ab	9.5 ab	10.0	53.6	23.8 b
GV41	4.8 a	79.2 a	10.0 a	10.7	57.3	27.7 a
*Significance*	^∗∗^	^∗^	^∗^	n.s.	n.s.	^∗^
Low N	2.9 b	69.0 b	8.5	10.0	60.7	24.3
Opt N	4.7 a	80.5 a	10.8	10.4	55.4	26.6
High N	4.2 a	72.7 ab	8.5	11.3	55.0	25.4
*Significance*	^∗∗^	^∗^	n.s.	n.s.	n.s.	n.s.
Low N-NoT	2.2 d	56.3 b	6.7 c	9.6	57.3 bc	21.8 b
Low N -T22	3.1 cd	71.9 a	9.1 ac	9.6	55.7 bc	23.3 b
Low N -GV41	3.4 cd	78.8 a	9.7 ab	10.7	69.0 a	27.7 a
Opt N-NoT	3.4 c	75.1 a	10.0 ab	10.8	61.7 ab	27.4 a
Opt N-T22	4.8 b	81.6 a	10.9 ab	9.4	50.6 c	24.6 ab
Opt N-GV41	5.9 a	84.8 a	11.5 a	11.0	53.7 bc	27.8 a
High N-NoT	3.0 cd	71.4 a	8.4 bc	12.4	61.2 ab	25.4 ab
High N-T22	4.4 b	73.0 a	8.5 bc	11.0	54.5 bc	23.4 b
High N-GV41	5.1 ab	73.8 a	8.7 bc	10.5	49.2 c	27.5 a
*Significance*	^∗∗^	*^∗^*	^∗^	n.s.	^∗^	^∗^

*Different letters within each column indicate significant differences between treatments according to LSD test (*P* < 0.05). n.s., non-significant differences, ^∗^significant differences at *P* ≤ 0.05 and ^∗∗^ at *P* ≤ 0.01.*

*Low N, non-fertilized conditions (low N availability) for both crops; Opt N, optimal N fertilization, amounting to 90 kg N ha^-*1*^ and 60 kg N ha^-*1*^ for lettuce and rocket, respectively; High N, supraoptimal N fertilization, amounting to 180 and 120 kg N ha^-*1*^ for lettuce and rocket, respectively; NoT, non-inoculated control; T22, *T. harzianum* strain T22; GV41, *T. virens* strain GV41.*

Neither *Trichoderma* inoculation nor N fertilization dose had a significant effect on PO_4_^3-^ concentration in rocket leaves (**Table 1**). The K^+^ concentration in rocket leaves was only affected by the interaction of the biostimulants with the N fertilization level, with the highest values recorded in the unfertilized treatment. Finally, the *Trichoderma* inoculation, averaged over N fertilization dose, affected the Ca^2+^ concentration in rocket leaf tissue which was 14% greater than non-inoculated and T22 inoculated plants (**Table 1**).

### Prokaryotic and Eukaryotic Populations in Soil

**FIGURE 4:**
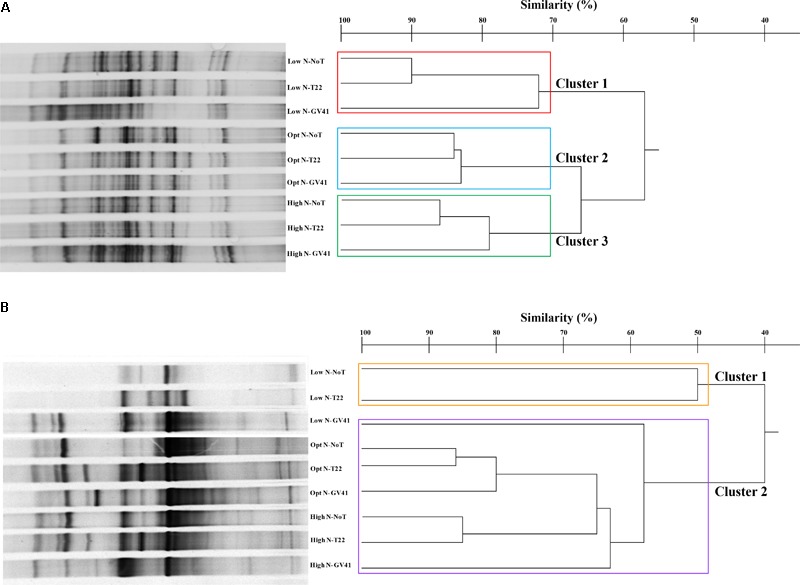
DGGE profiles and dendrogram showing the degree of similarity (%) of the PCR-DGGE profiles of the prokaryotes **(A)** and eukaryotes **(B)** in rhizo-soil samples from lettuce. Low N, non-fertilized conditions (low N availability); Opt N, optimal N fertilization, amounting to 90 kg N ha^-1^; High N, supraoptimal N fertilization, amounting to 180 kg N ha^-1^; NoT, non-inoculated control; T22, *T. harzianum* strain T22; GV41, *T. virens* strain GV41.

In general, the DGGE profiles of prokaryotes in the lettuce soil samples produced 21–27 bands (**Figure 4A**). Statistical analysis on the position and intensity of the bands allowed the classification of three major clusters clearly associated to the three N doses applied in the experiment: Cluster 1: Low N; Cluster 2: Opt N; Cluster 3: High N (**Figure 4A**). Clusters 2 and 3 demonstrated a similarity of 66%, while Cluster 1 was only 57% similar to the assembly of these two groups. It was interesting to note that within each of the major clusters delineated by the N levels, the subgroupings of the prokaryotes were always similar and determined by the *Trichoderma* applications. In fact, the GV41 inoculation greatly influenced the composition of prokaryotes in the lettuce rhizosphere, always indicating a separation of the populations from this treatment as a distinct group. In all nitrogen fertilization conditions, the prokaryotes from NoT and T22 inoculations paired to form sub-clusters at high similarity levels of 90%, 84%, and 86% (for Low N, Opt N, and High N, respectively). The NoT and T22 sub-clusters were less related to GV41, differentiating at 72%, 83%, and 79% similarity in Low N, Opt N, and High N conditions, respectively.

DGGE of the eukaryotic populations in the lettuce rhizosphere showed a diverse profile, that did not clearly correspond to the fertilization, or was not consistently associated to the *Trichoderma* treatments in the groupings formed (**Figure 4B**). Cluster analysis identified two major groups, with a similarity of 40%: Cluster 1 included Low N-T22 and Low N-GV41; whereas, Cluster 2 was comprised of Low N-NoT, Opt N-NoT, Opt N-T22, Opt N-GV41, High N-NoT, High N -T22, High N-GV41. The major Cluster 1, included the non-fertilized soils inoculated with T22 and GV41, that contained the lowest eukaryote biodiversity (eight bands); whereas the Cluster 2 grouped eukaryotes from the N-fertilized soils and Low N-NoT sample produced a number of bands ranging from 17 to 23.

The *Trichoderma* inoculations strongly influenced the composition of eukaryotic populations in the absence of nitrogen fertilization in lettuce whereby inoculations with T22 and GV41 formed a sub-cluster that was not highly comparable (50% of similarity). Instead, under both Opt N and High N a sub-cluster was observed that included NoT and T22 with a high level of similarity (85–86%), that was divergent from GV41 (**Figure 4B**).

**FIGURE 5:**
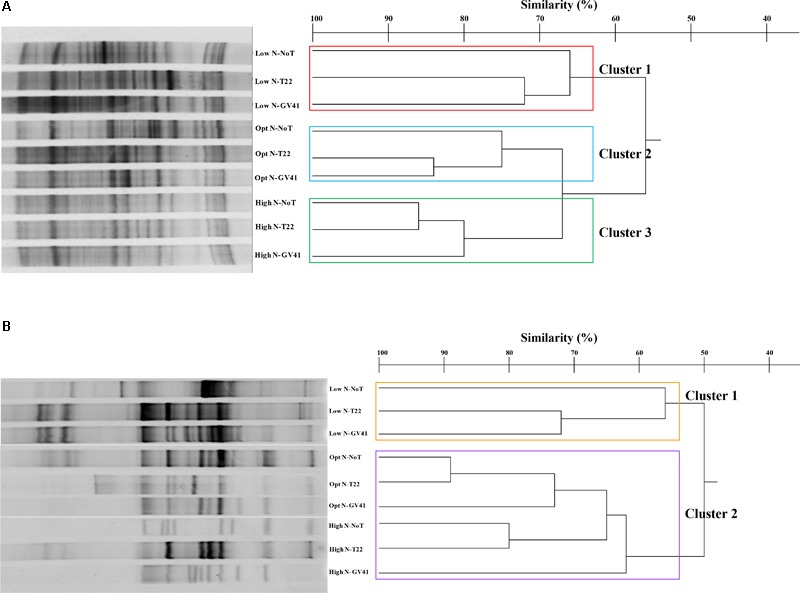
DGGE profiles and dendrogram showing the degree of similarity (%) of the PCR-DGGE profiles of the prokaryotes **(A)** and eukaryotes **(B)** in rhizo-soil samples from rocket. Low N, non-fertilized conditions (low N availability); Opt N, optimal N fertilization, amounting to 60 kg N ha^-1^; High N, supraoptimal N fertilization, amounting to 120 kg N ha^-1^; NoT, non-inoculated control; T22, *T. harzianum* strain T22; GV41, *T. virens* strain GV41.

The prokaryotic populations in rocket rhizo-soil samples exhibited 29–43 bands in DGGE profiles (**Figure 5A**). The cluster analysis of the DGGE identified three major clusters that grouped on the basis of nitrogen fertilization applied, similar to results observed in the rhizosphere of lettuce Clusters 2 and 3, with nitrogen treatments, grouping at a similarity level of 67%, while the Cluster 1 was only 56% similar to these groups. The effect of *Trichoderma* inoculations to the prokaryote populations was variable depending upon the N inputs. T22 and GV41 inoculations showed a similarity of 84% under optimal fertilization and 75% under no fertilization, always separating distinctively from the no biostimulant treatment (**Figure 5A**). Under High N, T22 and NoT formed a sub-cluster with a 86% similarity that was 80% similar to GV41.

Once again, the eukaryotic populations showed a different complex of DGGE patterns (11–26 bands) compared to prokaryotes, that was similar to that noted with lettuce (**Figure 5B**). Statistical analysis indicated two major clusters that separated at a similarity level of 50% into a group with low N conditions (Cluster 1: Low N-NoT, Low N-T22, Low N-GV41), and a grouping with the fertilized conditions (Cluster 2: Opt N-NoT, Opt N-T22, Opt N-GV41, High N-NoT, High N-T22, High N-GV41) (**Figure 5B**).

The effect of *Trichoderma* inoculation on eukaryotic populations of rocket rhizosphere was more marked than that observed with the prokaryotes. Both strains T22 and GV41 formed a sub-cluster at a similarity level of 72% under non-fertilized conditions, that was 56% similar to NoT. On the contrary, in both fertilization levels, a sub-cluster including non-inoculated and T22, grouped at 89% and 80% similarity for Opt N and High N, respectively, that separated them from GV41 at a similarities of 73% and 62% for Opt N and High N, respectively.

## Discussion

### Implications of *Trichoderma*-Based Biostimulant for Crop Productivity and Leaf Quality

The application of *Trichoderma*-based agricultural products in vegetable cropping systems is on the rise, stimulated by the increasing interest of growers, extension specialists, and scientists to improve crop productivity in a sustainable manner (Woo et al., 2014; Colla et al., 2015c; Lorito and Woo, 2015). A biostimulant effect was noted in the greenhouse experiment with lettuce, demonstrating that inoculations of *Trichoderma* significantly increased the yield in all fertilization conditions. In particular, the application of strain GV41 had the most positive effect on production under both low and optimal N availability.

On the other hand, in rocket, the inoculations with the same *Trichoderma* did not produce a generalized advantage in yield that was comparable to that observed in lettuce. The treatment with GV41 produced a significant increase in yield, but only under low N input fertilization. This demonstrates that the response of the two tested leafy vegetables to *Trichoderma*-based biostimulants is not global, but complex, depending upon the crop botanical family (*Asteraceae* versus *Brassicaceae*), as well as the *Trichoderma* strains. Our findings are in agreement with the results of several research groups who have demonstrated that the increase in plant growth after applications of *Trichoderma* depends on the plant species, and also the genotypes (varieties) (Baker, 1988; Ousley et al., 1994). For instance, Ousley et al. (1994) demonstrated that the inoculation with six strains of *Trichoderma* improved significantly the growth parameters of lettuce, whereas Baker (1988) who tested the same strains on radish and pea, reported a limited increase in crop performance. Furthermore, Tucci et al. (2011) showed that the inoculation of cultivated and wild tomato varieties with *T. atroviride* and *T. harzianum* improved crop performance in a genotype-dependent manner.

The increased plant growth and productivity of lettuce or rocket grown in varying nutrient conditions may involve several biological and chemical processes in the rhizosphere that are attributable to *Trichoderma* mechanisms of action, for example, the modification of soil nutrient availability or the modulation of root growth. Some *Trichoderma* species-strains are producers of secondary metabolites that are hormone-like, and the exudation of molecules with auxin-similar activity, small peptides or volatile organic compounds in the rhizosphere are known to have plant growth promotion effects (Vinale et al., 2008; Sofo et al., 2012; Pelagio-Flores et al., 2017). Specifically, in regards to the present investigations, *T. harzianum* strain T22 is not a noted producer of bioactive compounds that stimulate plant growth, however, applications of T22 in the rhizosphere is able to activate plant metabolic processes involving phytohormones (auxins/cytokinins) in treated plants, i.e., in cherries GiSeLa6, that improve above-ground plant and below-ground root system development (Harman, 2000; Sofo et al., 2012). Some *T. virens* are known to produce auxinic compounds that effect plant growth (Pelagio-Flores et al., 2017), but in the case of *T. virens* GV41 (used in the present study), this has not been observed, and this strain has been more noted as a producer of gliotoxin, a compound that may protect the plant due to its inhibitory effects on microbes that damage plant health (Vargas et al., 2014).

Interestingly, another factor that appears to have an important role on the *Trichoderma* stimulation effect of the plant is the regulation of pH in the rhizosphere by this fungus. *Trichoderma* can modify the acidity of the root environment that influences the plant response in the interaction with the fungus, relative to the production of active hormonal compounds in diverse genomic and functional processes (Sofo et al., 2012; Pelagio-Flores et al., 2017). Furthermore, recent work has demonstrated that root exudates produced by plants in response to biotic and abiotic stresses can act as chemoattractants to *Trichoderma*, thus regulating interactions with this microbe that result in beneficial plant effects (Lombardi et al., 2018).

Other mechanisms that *Trichoderma* uses that produce positive effects on plant growth include: plant disease control and induced resistance to pathogen attack; modulation of the root system architecture (total root length, density and branching) to increase area and absorption capacity for water and nutrient uptake; plus the assimilation/solubilization of macronutrients (P) and micronutrients (Fe, Mn, and Zn), possibly influenced by pH changes moderated by *Trichoderma* that can alter nutrient availability and uptake, thus boosting plant growth and yield (Altomare et al., 1999; Vinale et al., 2008; Hermosa et al., 2012; Sofo et al., 2012; Li et al., 2015; Pelagio-Flores et al., 2017). In our experiments, no effect on soil pH in the rhizosphere was recorded with the *Trichoderma* treatments whereby no significant difference was noted in soil samples analyzed at the beginning of the trial (average pH = 6.9 ± 0.2) and those analyzed at time of harvest for either crops (average pH = 7.1 ± 0.3). However, it is also possible that there was a temporal change in soil pH initially during the first weeks of crop growth that favored plant nutrient uptake, however, the measurements conducted during the experiment were not sufficient enough to detect this variation. Finally, *Trichoderma*, as a member of soil microbe community, has a role in the multitude complex of physical, chemical, and biological interactions that occur and greatly influence the agro-environment, and consequently effects crop health and productivity.

It is well established that *Brassicaceae* species, such as rocket, are noted for their negative effects on many soil microbes, bacteria, and fungi, due to the production of numerous inhibitory compounds such as glucosinolates that are released in the soil of the rhizosphere (Majchrzak et al., 2010; Ackroyd and Ngouajio, 2011; Szczygłowska et al., 2011). Further, the limited effect of *Trichoderma*-based biostimulants on rocket could be associated to several intrinsic preharvest factors, such as crop rotation, the N availability conditions, as well as the method of application of the inoculum, that may influence the establishment and colonization of these beneficial microbes in the rhizosphere. *Brassicaceae* are well known for their high phytoextraction ability, as proven by their frequent use as N catch-crops in cropping systems to limit nitrate leaching during the rainy season (Justes et al., 2012). Due to its high N uptake, rocket can be also used for biomonitoring of mineral N flush after fertilizer application (León Castro and Whalen, 2016). The better nutritional status (higher K^+^, Ca^2+^, PO_4_^3-^), as well as the higher N uptake recorded for rocket in our greenhouse trial supports this characteristic. It is possible that the high root-uptake capacity of rocket buffered the *Trichoderma* biostimulation in fertilized plots, a positive effect that was only noted in the non-fertilized treatment.

The differential beneficial effects of *Trichoderma* on the two tested crops could also be attributed to the length of the growing cycle (57 and 28 days for lettuce and rocket, respectively) in which the influence of the fungus on the plant is limited by the time of its interaction with the treated crop. The longer duration of the lettuce crop cycle also provides more time for root system development and diversification, influencing the ability of the microbial-based biostimulant to better colonize the crop rhizosphere (Alonso-Ramírez et al., 2014), thus increasing the efficiency of both *Trichoderma* biostimulants under different N availability conditions. On the contrary, the shorter growing cycle, such as that of rocket, may only result in biostimulation if the beneficial microbe has a higher performance (i.e., GV41), due to an ability to more rapidly colonize the rhizosphere under N limiting conditions. Furthermore, successful results with biostimulant depend not only on the crop rotation, strains and length of the growing cycle, but also on the method of application. In the present study, different modes of biostimulant application were used due to the different methods of initial crop establishment in the field, lettuce by transplant of seedlings and rocket by direct seeding. *Trichoderma* was applied directly to the roots of lettuce, thus permitting immediate contact of the fungal inoculum with the roots, whereas the application to rocket was by seed treatment, which is a reduced inoculant load and requires the development of a rhizosphere competent strain in order to colonize the developing root system. The different response of lettuce and rocket in terms of crop productivity to the *Trichoderma* inoculations in the present study confirmed the importance of the application method (Kleifeld and Chet, 1992; Brotman et al., 2013; Gupta et al., 2014; Mahmood et al., 2016).

Although the overall increase in leafy vegetables yield with *Trichoderma*-treatments is positive for the producers, enhancing product quality by microbial inoculation is a challenging and important issue to address in light of the growing interest of vegetable consumers, in particular those of fresh produce, to obtain products with a higher nutrient content (Kyriacou et al., 2016; Kyriacou and Rouphael, 2018). In the current study, the improvement in plant quality characteristics with *Trichoderma* inoculation was species-dependent since the beneficial effect was only observed on rocket. Interestingly, rocket plants treated with GV41 under optimal N and to a lesser extent under high N conditions, showed enhanced biosynthesis and accumulation of plant compounds, i.e., ascorbic acid, indicating that *Trichoderma*-based biostimulants can positively modulate plant secondary metabolism, as well as increase the phytochemicals that provide health benefits to the consumer (López-Bucio et al., 2015). Additional research is required in order to enhance understanding on the molecular and physiological mechanisms behind the capability of these plant beneficial microbes to increase phytochemicals that contribute to our well-being, and to select promising *Trichoderma* strains that are able to enhance the nutraceutical properties of horticultural commodities.

### Implications of *Trichoderma-*Based Biostimulant for Minimizing N-Input in Horticultural Systems

Nitrogen fertility management plays a key role in sustaining crop growth and ensuring food safety in horticultural-cropping systems by increasing N availability to both crops and soil microflora (Geisseler and Scow, 2014). N inputs must be adjusted accordingly, not only to crop requirements, but also to native soil fertility (Sanz-Cobena et al., 2017) in order to avoid an excess in N accumulation in vegetable produce such as nitrates. In addition, the N surplus unavailable to crops is prone to leaching to ground and surface water, that represents a serious environmental concern, especially in coarse soils with a high vulnerability to this phenomenon (Nitrates Directive 91/676/EEC). The soil in our experiment demonstrated a good fertility level due to its coarse texture, very low carbonate content and medium total C and N content. According to the empirical equation proposed by Remy and Marin-Lafleche (1974), this soil has a medium annual mineralization rate of 1.9% year^-1^ corresponding to an annual availability of 100 kg N ha^-1^ year^-1^ (calculated on a 0.40 m layer with a soil density of 1.2 Mg m^-3^). This calculation does not discriminate between different climatic conditions, nevertheless it is well known that in the Mediterranean region, the N mineralization peak under irrigated greenhouse conditions occurs during the spring–summer period (the same period of our experimental trial) when soil temperature and moisture are optimal for soil microbiota involved in the nitrification process. Analysis of crop N uptake in our trials confirmed this base level of nitrogen in the native soils, i.e., unfertilized plots, with values ranging from 70 to 100 kg N ha^-1^ for lettuce and rocket, respectively. In the absence of microbial-based biostimulants, application of fertilizers at the optimal (recommended) level increased crop yields in comparison to the unfertilized control, while excess fertilization did not modify crop growth and N uptake pattern. The nitrate content was not significantly influenced by either the biostimulant or the N applications, having values below the maximum threshold of nitrates (2,500 mg NO_3_^-^ kg^-1^ FW) imposed by Commission Regulation (EC) No 1881/2006 for lettuce as well as for rocket (6,000 mg NO_3_^-^ kg^-1^ FW in summer-grown rocket or 7,000 mg NO_3_^-^ kg^-1^ FW in winter-grown rocket).

According to these results, excessive N availability (due to fertilizer doses and soil native fertility) does not represent a problem to food safety for lettuce or rocket production in our study area, since the nitrate content in both leafy vegetables was below the maximum limit established by the European Commission. This result could be associated to the high nitrate-reductase activity of leafy vegetables grown in Mediterranean area during the spring–summer growing period, usually coupled with a high photosynthetic activity (Larios et al., 2001).

As shown by several authors the effect of mineral fertilization can be significantly lowered in soils with a high native fertility, leading to slight variations in plant response and nutrient uptake (Montemurro et al., 2006; Alluvione et al., 2013). This was also observed in the present experiments where no difference in soil mineral N availability was recorded between the unfertilized control (Low N plot) and fertilized treatments to the harvest of both lettuce and rocket. Our hypothesis is that the high soil organic matter mineralization rates occurring under greenhouse conditions increased native soil mineral N availability, buffering the effect of N fertilization, as the difference in N root uptake activity of the two crops and with *Trichoderma* biostimulation emerges especially in the unfertilized conditions. This was true for both lettuce and rocket whose N uptake significantly increased with GV41*-*based biostimulant in unfertilized plots, pushing up values to the same level of non-inoculated plants in fertilized plots. In addition, GV41 significantly increased N uptake in lettuce under Opt N fertilization, as well as in rocket in unfertilized conditions, highlighting that specific strains, i.e., *T. virens* strain GV41, can be applied to increase NUE and reduce N surplus in horticultural systems. From these results stems a very interesting opportunity for designing a lettuce-rocket crop rotation program aimed at managing N input–output resources. This objective could be achieved by performing an optimal N fertilization only on the first crop, in this case lettuce, combined with applications of the GV41 biostimulant to both crops. In this manner, the second vegetable production (non-fertilized rocket) could take advantage of the residual N fertility remaining in the soil from the previous crop, thus maintaining vegetable yields close to those of fertilized systems and minimizing the N leaching risk due to soil N surplus. It must be pointed out that, in order to be sustainable, this approach should be applied in high fertility soils, similar to that tested in our trial, or when a long-term strategy to maintain soil fertility is adopted (i.e., organic farming). Finally, the marked positive effect of *Trichoderma*-based biostimulants under Low N availability conditions highlighted that GV41 inoculation can be a viable strategy to increase yield and nutrient uptake of leafy crops in low fertility soils.

### Implications of *Trichoderma*-Based Biostimulants for Modulating Soil Microbial Communities

A culture-independent approach was employed to obtain a qualitative picture of the effect of the chemical and biological amendments on resident soil microbial community in the rhizosphere of lettuce and rocket. The cluster analysis demonstrated that prokaryotic populations were affected more by nitrogen fertilization levels than by the *Trichoderma* inoculations. Different nitrogen treatments can determine different plant mediated effects that induce changes in the associated microbial communities of the rhizosphere (Giagnoni et al., 2016). Li et al. (2016) reported that different levels of N significantly influenced the distribution and composition of the bacterial community in a long-term monoculture of lettuce, as well, N inputs can significantly alter microbial diversity in vegetable production systems. This is true for the regulation of N-fixing activity of soil microbiota, particularly at a transcriptional level, that is affected by factors such as the application of different nitrogen sources (Saraf et al., 2011). The metabolically expensive nitrogenase system of free-living diazotrophs is more sensitive to levels of ammonium rather than to nitrate, since the process is repressed when the cellular level of fixed nitrogen is sufficiently high (Ruppel and Merbach, 1995; Saraf et al., 2011). Application of N fertilizers can stimulate plant root exudation allowing a better utilization of nutrients by microbiota (Sørensen, 1997). The microbiota in agro-ecosystems may respond differently to N fertilization and this could lead to unpredictable results on N-fixing activity in the rhizosphere (Saraf et al., 2011). Recently the disturbance of some bacterial populations in response to chemical fertilization, especially those involved in the nitrogen cycling, is a phenomenon that has been well documented (Gupta et al., 2015; Fiorentino et al., 2016).

*Trichoderma* inoculations also affected the bacterial community structure in the rhizosphere of both lettuce and rocket, although the effect of the two microbial-based biostimulants differed according to vegetable crops and/or to N inputs. The composition of prokaryotic populations was more influenced by *T. virens* GV41 than by *T. harzianum* T22 in the lettuce rhizosphere, whereas a clear separation was observed in the GV41 treated soils, in all nitrogen fertilization conditions. However, in the rocket rhizosphere, the same distribution of prokaryotes was detected only under High N-fertilization with GV41; while at both Low N and Opt N inputs, soils were strongly affected by both microbial biostimulants. Gupta et al. (2016) observed significant changes of the qualitative profiling of microbial communities in biological-treated rhizosphere samples, indicating a noticeable impact of bacterial and fungal inocula on native microbial populations.

The combination of chemical and biological amendments exerted different effects on the native eukaryote communities, depending upon the plant species. It is known that diverse crops and plant compounds can strongly affect soil microbial communities, and these factors are the primary players that have a direct impact on soil microbial dynamics (Larkin, 2008). Similarly, the main driver for fungal population diversity was the fertilization, regardless of N levels, since a clear separation was observed among non-treated and N treated soils for both leafy vegetables, indicating that fungi were less sensitive to the N inputs than bacteria. Moreover, *Trichoderma* inoculation influenced the eukaryotic population of the rhizosphere in the non-fertilized treatment, and this effect was more pronounced especially in lettuce since the fungal biodiversity was strongly reduced. Overall, results emphasized that the eukaryotic communities were affected differently by soil fertilization than the prokaryotic populations. The differences in native bacterial and fungal rhizosphere populations could be due to the fact that bacteria are generally more sensitive to any environmental changes since they have a much shorter generation time than fungi, and therefore can respond more quickly to soil amendments (Lazcano et al., 2013).

The highly complex prokaryotic DGGE patterns compared to eukaryotic profiles in rocket soil samples highlighted that the rhizosphere was mainly dominated by bacteria, as supported by previous studies (Bardgett, 2005; Gupta et al., 2016). However, under optimal fertilization, an increase in fungal biodiversity was observed probably due to the fact that the optimal N fertilization exerted a growth and activity stimulation effect on resident fungal populations.

## Conclusion

The increased pressure on vegetable farmers to maximize crop productivity, but at the same time limit the use of synthetic N fertilizers, represents an important challenge for plant scientists in research to develop novel strategies to secure agricultural production in a sustainable manner (by increasing NUE). *T. virens* GV41 was proven to be the best performing microbial-biostimulant in terms of crop growth and nutrient uptake on both lettuce and rocket, demonstrating the differential effect of different fungal inoculants on crop production. This biostimulant increased fertilizer N use efficiency of lettuce, favored the uptake of native soil N by both lettuce and rocket, and also improved N uptake by roots under low N availability conditions. The application of *Trichoderma*-based products to leafy vegetable cropping systems can be considered to increase crop yield, nutrient use efficiency and crop quality (i.e., mineral and ascorbic acid content). The effect of the biostimulants can significantly vary depending on the duration of the crop cycle, method of inoculant application, and the specific root uptake efficiency of the plant species. Additional experiments should be performed in diverse pedoclimatic conditions in order to further investigate the important issues that need to be addressed for developing efficient crop management practices. Moreover, it is important to determine the impact that such inoculants may have on the native microbial populations and investigate the manner that these applications may act, not only as direct biostimulants of crops, but also as beneficial components of the plant microbiome to improve productivity. The findings of this study can have a positive impact in the design of horticultural crop rotation systems aimed at minimizing N inputs, reducing the consequent risks to the environment and establishing sustainable agriculture.

## Author Contributions

NF defined the experimental protocol, carried out soil and vegetable samplings, coordinated crop N analyses, performed the data elaboration and statistical analyses of crop performance (yield and N uptake), and was significantly involved in writing the whole manuscript. YR defined the experimental protocol, coordinated the ion and ascorbic acid analyses, performed the data elaboration and statistical analyses of crop quality, and was significantly involved with GC (for the agronomic part) in writing the whole manuscript. VV and IR carried out the molecular characterization (DGGE) of soil microorganisms, carried out data elaboration, and drafted the manuscript for this part. OP coordinated microbiological (DGGE) analyses and drafted the manuscript for this part. SW contributed to the setting-up of the experiment, was involved in results interpretation of microbiological data, and in writing of the manuscript for this part. ADR and LG carried out the experiments as well as soil and vegetable samplings, performed soil and plant analyses, and the first elaborations of agronomic data. NL prepared *Trichoderma*-biostimulants and carried out plant inoculations, and she collaborated in manuscript preparation for the microbiological section. MN performed the microbial counts and statistical analysis, and he collaborated in manuscript preparation.

## Conflict of Interest Statement

The authors declare that the research was conducted in the absence of any commercial or financial relationships that could be construed as a potential conflict of interest.

## Footnotes

1 http://agri.istat.it

2 https://www.iso.org/standard/38924.html

3 https://www.iso.org/standard/21870.html

## Supplementary Material

FIGURE S1 Photos showing representative lettuce and rocket plants as affected by different N doses and *Trichoderma* inoculation. Low N, non-fertilized conditions (low N availability) for both crops; Opt N, optimal N fertilization, amounting to 90 kg N ha^-1^ and 60 kg N ha^-1^ for lettuce and rocket, respectively; High N, supraoptimal N fertilization, amounting to 180 and 120 kg N ha^-1^ for lettuce and rocket, respectively; NoT, non-inoculated control; T22, *T. harzianum* strain T22; GV41, *T. virens* strain GV41.
