# Supplementary figures and images for: Trichoderma-Based Biostimulants Modulate Rhizosphere Microbial Populations and Improve N Uptake Efficiency, Yield, and Nutritional Quality of Leafy Vegetables
Source: Front Plant Sci. 2018 Jun 5;9:743. doi: 10.3389/fpls.2018.00743 (PMC5996573; doi:10.3389/fpls.2018.00743)

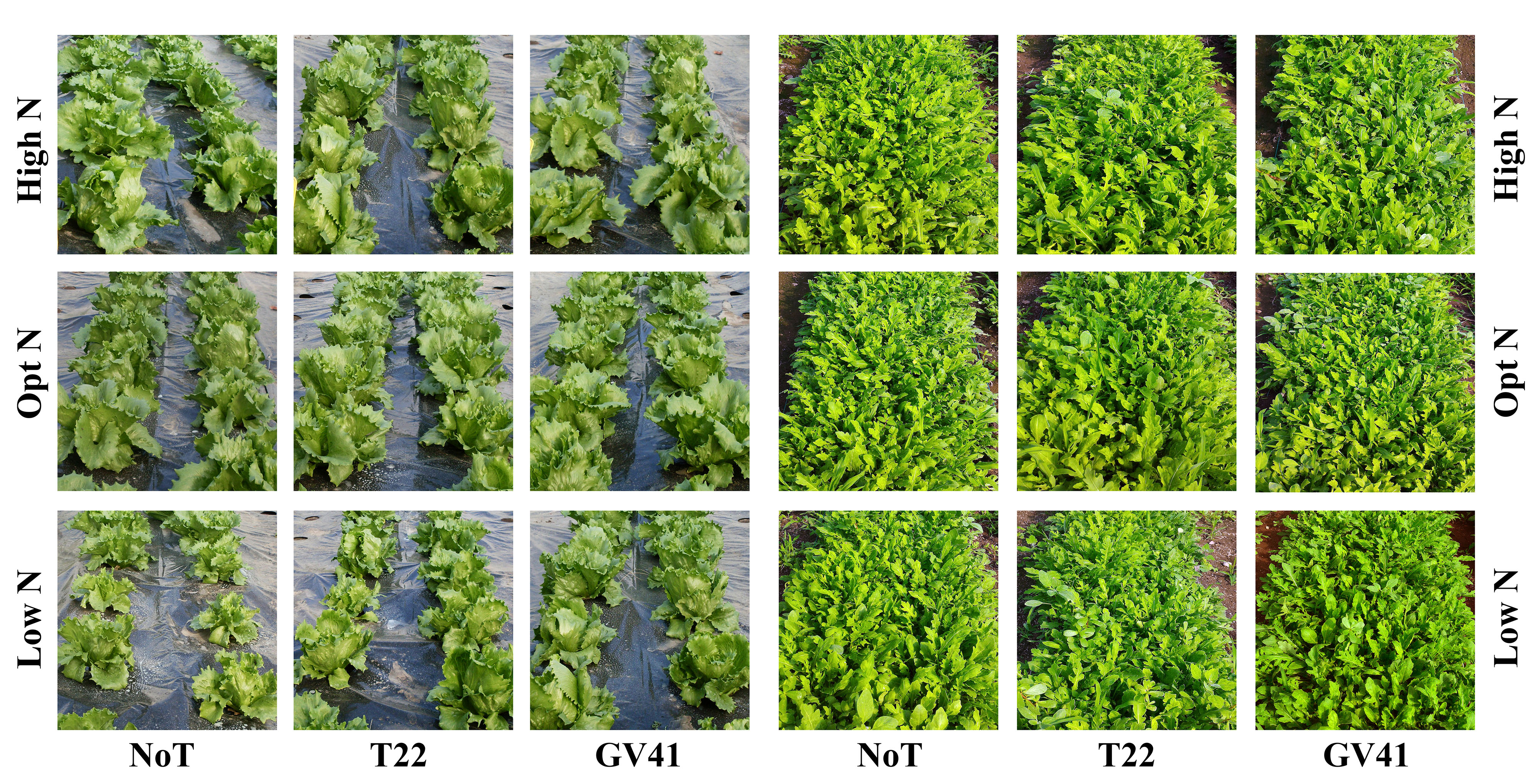

Supplement: FIGURE S1 — Photos showing representative lettuce and rocket plants as affected by different N doses and Trichoderma inoculation. Low N, non-fertilized conditions (low N availability) for both crops; Opt N, optimal N fertilization, amounting to 90 kg N ha-1 and 60 kg N ha-1 for lettuce and rocket, respectively; High N, supraoptimal N fertilization, amounting to 180 and 120 kg N ha-1 for lettuce and rocket, respectively; NoT, non-inoculated control; T22, T. harzianum strain T22; GV41, T. virens strain GV41. [file Image_1.JPEG]
